# Decoding Pathogenic Mutational Landscapes in Alzheimer′s Disease Through Integrated Transcriptomics

**DOI:** 10.1155/humu/6627566

**Published:** 2026-05-12

**Authors:** Wan Ma, Fenfang Zhou, Huaying Cai, Ling Fang

**Affiliations:** ^1^ Affiliated Mental Health Center & Hangzhou Seventh People′s Hospital, Zhejiang University School of Medicine, Hangzhou, Zhejiang, China, zju.edu.cn; ^2^ Department of Neurology, Sir Run Run Shaw Hospital, Zhejiang University School of Medicine, Hangzhou, Zhejiang, China, zju.edu.cn

**Keywords:** Alzheimer′s disease (AD), inflammatory response, mutations, transcriptomic alterations, TUBB2A

## Abstract

Alzheimer′s disease (AD) is increasingly understood as a disorder driven not only by amyloid and tau pathology but also fundamentally shaped by underlying genetic mutations. By integrating multiple AD gene expression datasets with machine learning approaches—including random forest, XGBoost, LASSO, and SVM—we identified 172 differentially expressed genes, with TUBB2A, RTN4, and YWHAZ emerging as top mutation‐associated hub genes. Critically, TUBB2A not only exhibited strong diagnostic potential (AUC = 0.822) but also harbored somatic mutations in our patient cohort, directly linking mutational events to disease manifestation. Unsupervised clustering revealed two distinct AD subtypes: one marked by widespread early gene overexpression and another (Cluster 2) dominated by endoplasmic reticulum stress—likely reflecting divergent mutational landscapes. Pseudotemporal trajectory analysis demonstrated a continuous progression from normal samples to Cluster 2, suggesting that a pivotal mutational event may initiate this transition and accelerate disease progression. These findings underscore the central role of somatic and germline mutations—particularly in TUBB2A—in AD pathogenesis. Our study strongly supports a paradigm shift toward mutation‐centric biomarker development and advocates for SNP‐based strategies to enable early diagnosis and personalized therapeutic interventions tailored to individual mutational profiles.

## 1. Introduction

Alzheimer′s disease (AD), which is the most common type of neurodegenerative disorders [1, 2], is a slow progressing degenerative brain disease that affects cognitive abilities such as memory, loss of mental capacity, and brain cell death [3]. In addition to classical pathology hallmarks, such as accumulation of *β*‐amyloid (A*β*) plaques and phospho‐tau tangles [4–6], the increasing amount of evidence indicates that such separate routes cannot explain the full extent and variability in the phenotypic expression of the diseases, where recent studies highlighted that inflammation and immunological dysfunction play a key role in disease pathogenesis [6, 7].

There is mounting evidence for the presence of chronic neuroinflammation as a defining feature across AD progression [8, 9]. It has been shown that overactivation of microglia induces secretion of inflammatory cytokines followed by synapse loss and neuronal death [9]. In addition, aberrant immunological processes such as increased regulatory T cells (Tregs) and decreased natural killer (NK) cell function have been reported [8–12] to play a role in changing the immune profile of AD. This evidence supports the hypothesis that AD is an inflammatory disease with an imbalance between proinflammatory and anti‐inflammatory responses, which is far more extensive than a simple amyloid‐beta and tau proteins pathology model [13].

However, how inflammation and immunological dysregulation are involved in AD is still not fully elucidated [2, 14–16]. However, most research only considers one cohort of patients without exploring multiple datasets systematically, which greatly limits our ability to discover robust biomarkers that can accurately identify diseases or distinguish subtypes at the molecular level.

To overcome the limitations of previous studies, in this study, we comprehensively combined various cohorts′ microarray data for a series of analyses such as differential expression analysis and weighted gene coexpression network analysis (WGCNA) method so that we could find hub genes associated with inflammation. We then employed machine learning techniques in order to assess the utility of each for medical diagnosis. Moreover, we characterized the immune cell map of AD, conducted gene expression–based classification by nonnegative matrix factorization (NMF), and performed temporal progression modeling to describe how the disease pathology progresses from an inflammatory state into one driven by endoplasmic reticulum (ER) stress. Together, our results offer new insights on the inflammatory and immunologic pathways involved in AD, providing potential candidate biomarkers as well as individualized intervention strategies for early diagnosis and precision medicine.

## 2. Methods

### 2.1. Data Collection and Preprocessing

AD‐related transcriptome data are downloaded from a public database, specifically for Gene Expression Omnibus (GEO). In order to reduce possible differences due to various research groups, we used the “sva” package installed on R software to correct for batch effects, and we analyzed data quality using PCA, which allowed for validating dataset comparability. Clinical data were normalized where possible.

### 2.2. Differential Expression and Functional Enrichment Analyses

The limma package was used to identify differentially expressed genes (DEGs) between the AD and control samples, using cutoffs of *p* value < 0.1 and absolute log2 fold change > 0.58. To study the underlying biology, Gene Ontology (GO) enrichment analysis was carried out for the biological process, cellular component, and molecular function categories in total. Furthermore, we performed KEGG pathway enrichment analyses for determining significantly enriched pathways.

### 2.3. WGCNA

The weighted correlation network analysis (WGCNA) package in R language was used for building a weighted gene coexpression network. The suitable soft threshold power is chosen so that the network has approximately a scale‐free topology property; genes are grouped together by calculating their topological similarity coefficients into different modules; finally, the relationship between each module′s characteristic gene expression and AD‐related clinical features is analyzed. Having analyzed relationships of the module eigengenes with AD phenotypes, we added genes in those significantly correlated modules into known gene sets related to AD as well as inflammation for identifying hub genes.

### 2.4. Machine Learning Modeling and Validation

To identify significant genes, we applied several ML techniques such as random forest (RF), extreme gradient boosting (XGBoost), logistic regression (LR), least absolute shrinkage and selection operator (LASSO), and support vector machine (SVM). The selection of important features was done using LASSO and RF methods, where the intersection of genes was considered to be a set of overlapping genes for both techniques and thus robust core biomarkers. For the primary cohort as well as for the independent validation cohort, diagnostic accuracy was evaluated by drawing ROC curves, where AUC is used as one of the evaluation metrics for measuring the performance of models.

### 2.5. Immune Infiltration Analysis

The measurement of immune pathway activity such as CCR signaling, interferon (IFN) type I response, Tregs, and macrophage‐related signatures was assessed using the single‐sample gene set enrichment analysis (ssGSEA) method; immune infiltration scores of multiple immune cells were calculated with CIBERSORT and XCell algorithm, respectively. To further investigate the underlying biological meaning behind such immunological differences, we re‐applied ssGSEA for quantifying pathway activation state regarding immunity, including pathways of CCR signaling, IFN type I response, regulation of T cells, and macrophages markers. Differential expression was tested for significance using pairwise *t*‐test statistics between AD cases versus controls; data are visualized in heat maps.

### 2.6. Molecular Subtyping

We used the NMF algorithms to partition patients who were diagnosed as ADs in various subtypes based on their microarray data. We selected *k* using the following criteria: data distribution shapes, and the silhouette width index (SWI). Next, we investigated the infiltration pattern of each subtype based on the known subtypes with a bioinformatics method including CIBERSORT, XCell, and ssGSEA.

### 2.7. Pseudotime Trajectory Analysis

#### 2.7.1. Transcriptional Temporal Dynamics of AD Progression

In order to explore the temporal change during AD progression, we calculated pseudotemporal developmental trajectories by Monocle2. We focused on the genes that are related to neurotransmission and immune response, cell cycle, inflammation, apoptosis, transcriptional regulation, DNA damage, DNA repair, translation and protein synthesis, and oxidative stress pathways. These genes were annotated on the trajectories for understanding the underlying biological processes in early/middle/late stage of the disease.

### 2.8. ELISA Experiment

In ELISA, the first operation is to coat the microplate with a target antibody by placing it in an environment where this antibody can bind. Following rinsing operations to wash away excess reagents, a specific blocker reagent for reducing the background signal is added, followed by further cleaning of the plate, and finally, the addition of the test substances as well as calibrators, wherein allowing said target molecule(s) contact with said immobilised antibody (antibodies). After this incubation time, the plate is rinsed, and then, an antibody conjugated to an enzyme is added. Following a final rinse, a chromogenic substrate for signal generation and a stopping reagent is added, after which the reaction product′s absorbance was measured by a plate reader.

### 2.9. The TUBB2A mRNA Expression Level

TUBB2A mRNA levels were quantified using quantitative real‐time PCR (qRT‐PCR). Total RNA was extracted, DNase‐treated, and reverse‐transcribed into cDNA. The qPCR reaction mixture, containing cDNA, specific primers, and SYBR Green Master Mix, was prepared on ice and analyzed on an automated thermal cycler. The cycling protocol consisted of an initial denaturation at 95°C, followed by 40 cycles of denaturation, annealing, and extension. Product specificity was confirmed via melting curve analysis. Relative expression levels were calculated using the $2^{-\Delta \Delta C_{t}}$ method, normalized to an internal reference gene.

### 2.10. Statistical Analysis

All statistics and data analysis was performed in R. Significance is set at two‐sided *p* < 0.05 unless otherwise noted. All figures were generated using “ggplot2,” “pheatmap,” and other related graphic packages from the R environment.

## 3. Result

### 3.1. Data Preprocessing and DEG Identification

**Figure 1:**
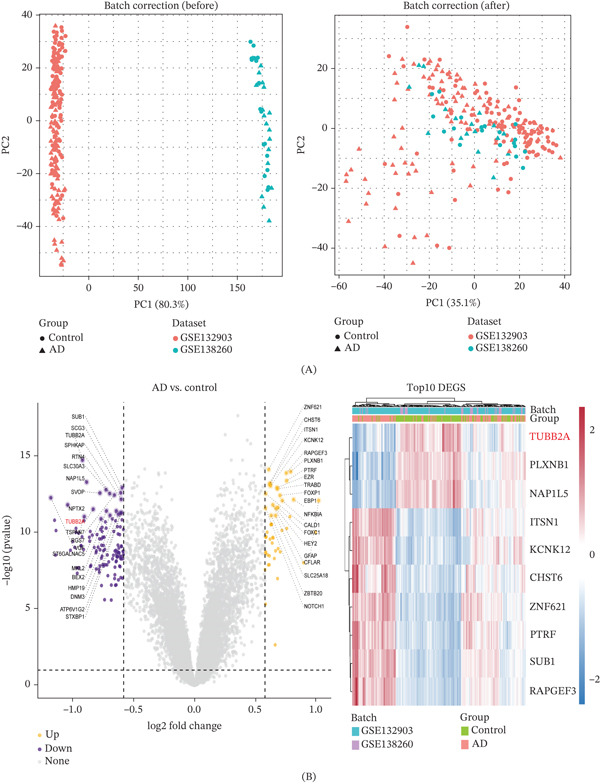
Identification of differentially expressed genes (DEGs) in Alzheimer′s disease. (A) Principal component analysis (PCA) demonstrated the effectiveness of batch correction, reducing technical heterogeneity across datasets. (B) Volcano plot showing DEGs between AD and control groups (yellow: upregulated; purple: downregulated; gray: nonsignificant). Representative upregulated genes include ZNF621 and SUB1, whereas TUBB2A and ITSN1 were significantly downregulated. Heat map of DEGs displaying distinct expression patterns between AD and controls, with TUBB2A downregulated and PLXNB1 upregulated in AD.

PCA confirmed that batch correction successfully mitigated technical artifacts, thereby aligning the corrected dataset with the biological signatures of AD (Figure 1A). Using the limma package (thresholds: *p* < 0.1, |logFC| > 0.58), we identified 49 upregulated and 123 downregulated DEGs. Visualization via volcano plots and heat maps (Figure 1B) not only highlighted specific dysregulated genes (e.g., upregulated ZNF621 and SUB1) but also underscored the global expression divergence between AD patients and controls. Crucially, we observed a marked downregulation of TUBB2A concomitant with an upregulation of PLXNB1, implicating both genes in AD pathogenesis. GO enrichment analysis revealed that these DEGs are predominantly associated with synaptic dysfunction. Key enriched terms included “synaptic vesicle traffic” and “neurotransmitter release cycle” (biological process); “secretory” and “presynaptic vesicle membranes” (cellular component); and “protein complex binding” alongside “actin cytoskeleton” organization (molecular function) (Figure S1B). Collectively, these data point to a central role for synaptic transmission dysregulation in AD. Complementing these findings, KEGG pathway analysis identified significant enrichment in neurotransmission‐related pathways, specifically “inhibitory neurotransmission” and “neurotransmitter vesicle dynamics.” Moreover, the integration of immune‐related pathways, such as opioid dependence and HIV‐1 infection, suggests a mechanistic link between compromised neural signaling and immune dysregulation in AD progression (Figure S1A).

### 3.2. Coexpression Network Construction and Hub Gene Identification

**Figure 2:**
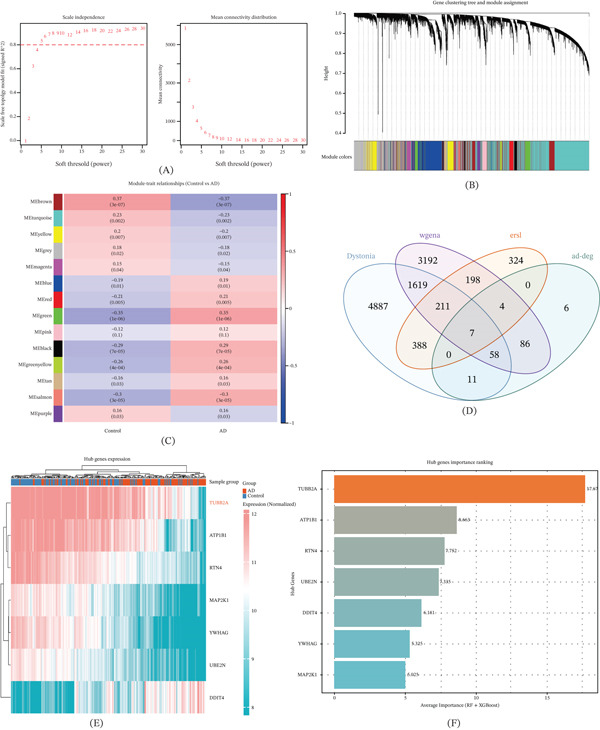
Coexpression network and hub gene identification. (A) Scale‐free topology fit and mean connectivity used to determine the optimal soft‐threshold power (*β* = 6). (B) Hierarchical clustering identified 14 coexpression modules. (C) Correlation analysis revealed that the MEbrown module was strongly negatively correlated with AD. (D) Venn diagram showing the intersection of DEGs, WGCNA hub genes, endoplasmic reticulum stress–related genes, and dystonia‐associated genes from GeneCards, yielding seven overlapping candidates. (E) Heat map of the seven overlapping genes across AD and control samples. (F) Machine learning–based importance ranking (random forest and XGBoost) highlighted TUBB2A as the most critical hub gene, followed by ATP1B1 and RTN4.

To construct a robust gene coexpression network adhering to scale‐free topology criteria, we evaluated the goodness‐of‐fit and mean connectivity across a range of soft‐thresholding powers (Figure 2A). An optimal power of *β* = 6 was selected, yielding a high scale‐free fit index. Subsequent hierarchical clustering delineated 14 distinct coexpression modules (Figure 2B). Among these, the MEbrown module displayed a pronounced negative correlation with AD clinical traits, highlighting its potential relevance to disease pathology (Figure 2C). Through a multisource integration strategy combining AD‐related DEGs, ER stress markers, dystonia‐associated genes (GeneCards), and WGCNA hub genes, we converged on seven high‐confidence candidate genes: TUBB2A, ATP1B1, RTN4, UBE2N, MAP2K1, DDIT4, and YWHA. Heat map visualization confirmed significant differential expression of these candidates between AD and control cohorts (Figure 2D). To further prioritize these targets, we applied ensemble machine learning models (RF and XGBoost) for feature importance ranking. This analysis unequivocally identified TUBB2A as the top‐ranked hub gene, with ATP1B1 and RTN4 emerging as the next most influential features (Figure 2E,F).

### 3.3. Machine Learning Validation and Diagnostic Value

**Figure 3:**
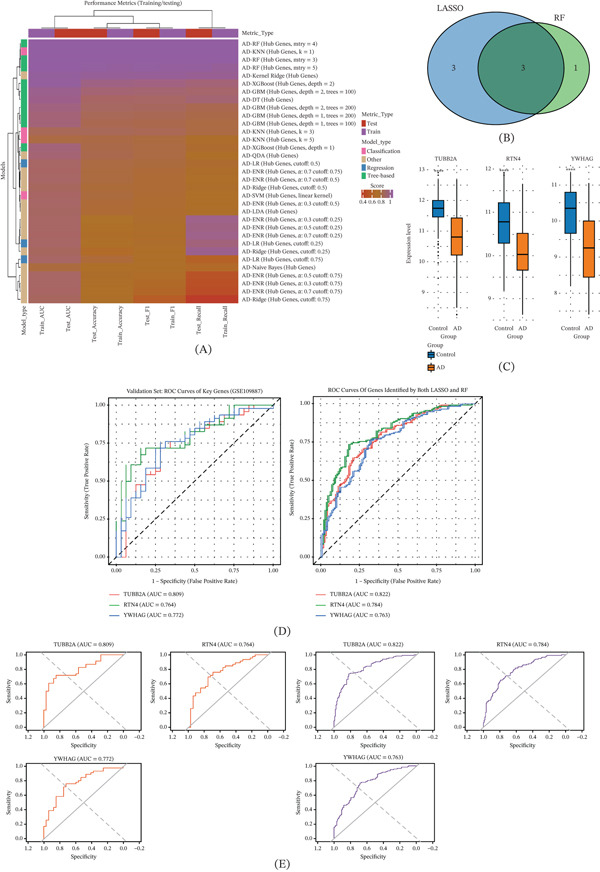
Machine learning validation and diagnostic performance of hub genes. (A) Schematic of machine learning model construction (RF, XGBoost, LR, and SVM). (B) Venn diagram showing the intersection of candidate genes identified by LASSO and RF, resulting in three hub genes (TUBB2A, RTN4, and YWHA). (C) Boxplots showing significantly lower expression of TUBB2A, RTN4, and YWHA in AD compared with controls. (D–E) ROC curves demonstrating diagnostic performance in training and validation datasets. TUBB2A exhibited the highest AUC (0.822 in training; 0.809 in validation), confirming its robustness as a diagnostic biomarker.

To discover the significant biomarker several machine learning methods have been used including RF classifier, XGBoost, LR, and SVMs (Figure 3A). Once we had built all of the above machine learning methods, a combination method using LASSO and RF were used for feature selection, which finally got three stable genes (TUBB2A, RTN4, and YWHA) (Figure 3B–C). In gene expression analyses, we found significantly lower levels of expression for all these candidate genes among AD patients compared with controls. The diagnostic accuracy was evaluated using the area under the receiver operating characteristic (ROC) curves and yielded good values, where we obtain a value of area under the ROC curve (AUC) as 0.822, 0.784, and 0.763, respectively, for TUBB2A, RTN4, and YWHA, which is above the threshold of clinical significance at 0.7. Notably, we verified this result on another dataset as a validation set (GSE109887) and found similar results of AUCs, thus further supporting the validity and translatability of these markers into a clinic setting for neurodegenerative diseases (Figure 3D–E).

### 3.4. Immune Infiltration Characteristics and Immune‐Related Gene Signatures in AD

To delineate the landscape of immune cell infiltration in AD, we applied both XCell and CIBERSORT algorithms. Comparative analysis revealed significant compositional shifts in AD tissues relative to healthy controls, most notably a marked expansion of Tregs and altered dynamics of resting and activated NK cells (Figure 4A,B). These data point to a concurrent dysregulation of immunosuppressive circuits and innate immunity, fostering a dysfunctional immune microenvironment in AD.

Figure 4Immune infiltration profiles and immune‐related gene expression in AD. (A) Immune cell infiltration estimated using XCell. (B) Immune composition analysis using CIBERSORT. (C–D) ssGSEA scores showed upregulation of CCR signaling and type I interferon response, along with downregulation of Treg‐ and macrophage‐associated pathways in AD. (E) Heat map clustering based on immune features separated AD samples (yellow) from controls (blue). (F) Expression analysis revealed significant upregulation of immune checkpoint gene PDCD1 and inflammatory gene LGALS3, and downregulation of cytotoxic marker CD8A in AD.

**Figure:**
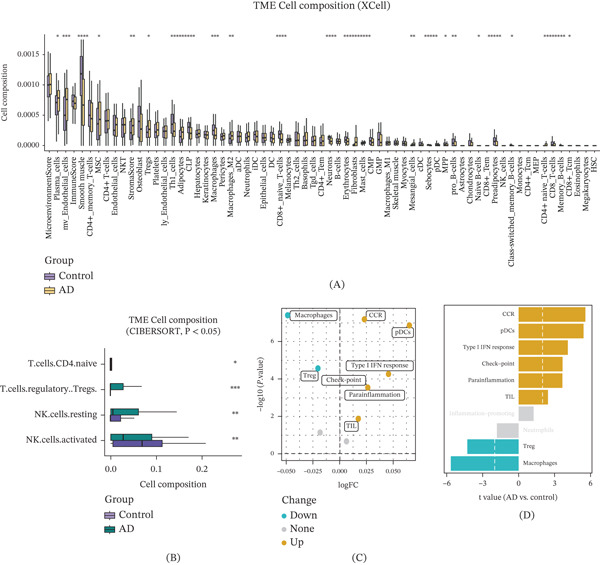
(A)

**Figure:**
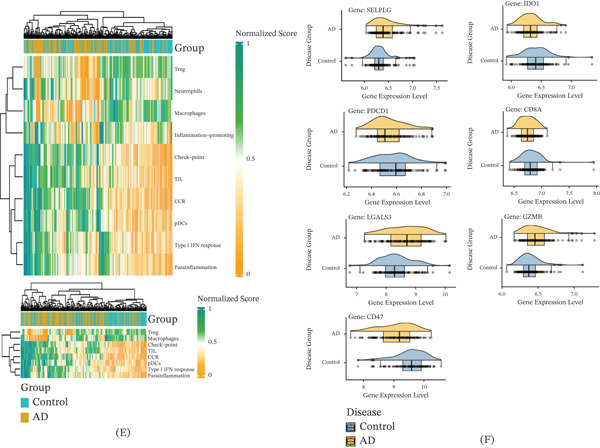
(B)

Complementing these findings, ssGSEA highlighted distinct functional disparities in immune traits. AD subjects exhibited hyperactivation of proinflammatory pathways, specifically the CCR signaling cascade and type I IFN response. In stark contrast, pathways governing Treg function and macrophage activity were significantly abrogated (Figure 4C). This dichotomy suggests a pathological skewing toward a proinflammatory state with compromised anti‐inflammatory resolution. Quantitative profiling corroborated these trends (Figure 4D), demonstrating elevated scores for CCR signaling, plasmacytoid dendritic cells (pDCs), and type I IFN responses in AD, alongside suppressed Treg and macrophage signatures. Unsupervised hierarchical clustering of these immune‐related DEGs robustly segregated samples into distinct “AD” and “control” cohorts (Figure 4E).

At the molecular level, this immune dysregulation was mirrored by the differential expression of key checkpoint and effector genes. AD cases showed significant upregulation of the immune checkpoint gene PDCD1 and the inflammatory mediator LGALS3, whereas CD8A, a critical marker of cytotoxic T‐cell function, was markedly downregulated (Figure 4F). Statistical validation confirmed these trends, underscoring a systemic disturbance in immune gene regulation. Collectively, the upregulation of inhibitory checkpoints and inflammatory drivers, coupled with the loss of cytotoxic potential, highlights a mechanism of immune exhaustion and chronic inflammation that may drive AD pathogenesis.

### 3.5. Molecular Classification, Immune Features, and Trajectory Analysis

NMF robustly stratified AD into two distinct molecular subtypes (*k* = 2), validated by high cophenetic correlation, clear t‐SNE separation, and consistent consensus clustering (Figure 5A,B,D). Transcriptomic and immune deconvolution analyses revealed a sharp dichotomy: Subtype 1 (C1) exhibits a proinflammatory milieu enriched in M1 macrophages and activated NK cells, whereas Subtype 2 (C2) is characterized by an immunosuppressive environment dominated by Tregs and quiescent NK cells (Figure 5C,E,G). Pseudotemporal trajectory analysis ordered samples along a Control right arrow C1 right arrow C2 axis, showing progressive upregulation of neuronal genes (e.g.,TUBB2A and RTN4) and ER stress markers (e.g.,DDIT4) (Figure S2). Crucially, we interpret the C2 subtype not merely as a stage of advanced pathology, but as a distinct stress‐adaptive or compensatory state. This transition mirrors the clinical shift from early focal tau deposition (Braak Stages I–III, corresponding to C1′s inflammatory phase) to widespread neocortical involvement (Braak Stages V–VI, corresponding to C2′s immune‐exhausted phase). The pronounced ER stress in C2 likely represents an initial protective unfolded protein response (UPR) against accumulating A*β* and tau burden; however, in the presence of underlying somatic mutations (e.g., in TUBB2A), this compensatory mechanism may eventually become maladaptive. Thus, C2 represents a critical tipping point where adaptive stress responses fail, suggesting that therapeutic strategies for this subgroup should target proteostasis restoration rather than solely suppressing inflammation.

Figure 5Molecular subtyping of AD and associated immune landscapes. (A) t‐SNE visualization showing clear separation of two molecular subtypes (C1: orange, C2: blue). (B) Consensus heat map confirming clustering stability. (C) Volcano plot of subtype‐specific DEGs (yellow: upregulated, purple: downregulated). (D) Evaluation metrics (cophenetic coefficient, dispersion, and silhouette score) supported *k* = 2 as the optimal number of clusters. (E, G) Immune infiltration analysis demonstrated that subtype C1 was enriched in T follicular helper cells, M1 macrophages, and activated NK cells, whereas subtype C2 was enriched in Tregs, resting NK cells, and M0 macrophages. (F) Boxplots illustrating subtype differences in Tfh cells, macrophages, and smooth muscle cells.

**Figure:**
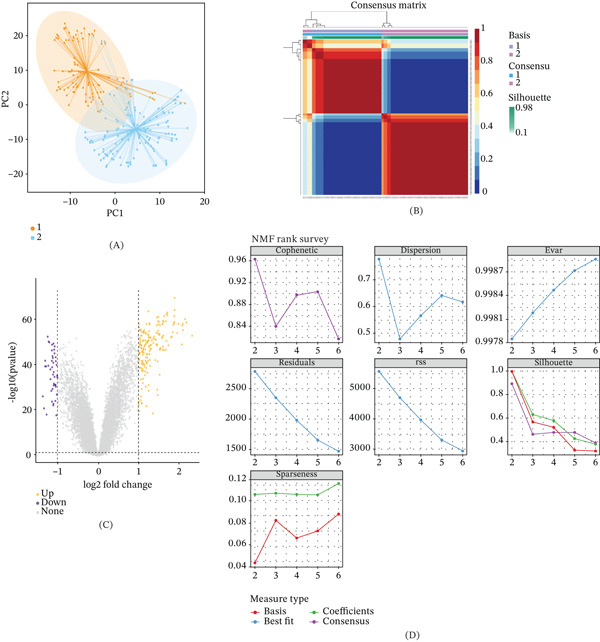
(A)

**Figure:**
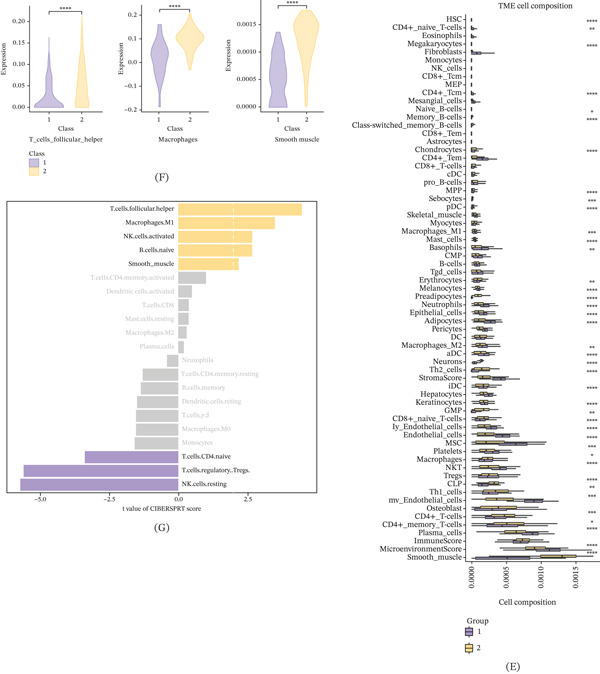
(B)

### 3.6. Elevated TUBB2A Expression Results in Decreased Secretion of Proinflammatory Cytokines

**Figure 6:**
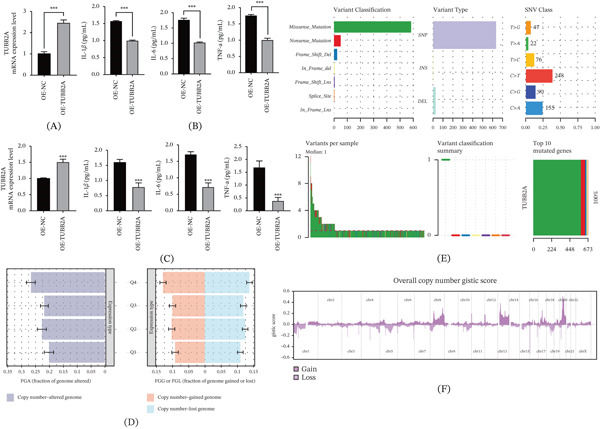
(A, C) Quantitative real‐time PCR confirmed that the mRNA expression level of TUBB2A was markedly increased in SH‐SY5Y and primary cells of mice cells transfected with the overexpression vector (OE‐TUBB2A) compared with the negative‐control group (OE‐NC) (∗∗∗*p* < 0.001). (B, C) Enzyme‐linked immunosorbent assay (ELISA) showed that the secretion of proinflammatory cytokines—including IL‐1*β*, IL‐6, and TNF‐*α*—was significantly decreased in the OE‐TUBB2A group relative to OE‐NC (D, E, F)Mutation status of TUBB2A (∗∗∗∗*p* < 0.0001; ∗∗∗*p* < 0.001).

To elucidate the functional role of TUBB2A in the AD microenvironment, we first established an overexpression model. qPCR analysis confirmed a robust upregulation of TUBB2A mRNA levels in the engineered cells compared with controls in SH‐SY5Y and primary cells of mice cells (Figure 6A,C). Functionally, this overexpression resulted in a significant suppression of key proinflammatory cytokines, including IL‐1*β*, IL‐6, and TNF‐*α*, as quantified by ELISA in independent replicates (Figure 6C,B), suggesting a potential neuroprotective capacity of TUBB2A against inflammatory stress. Beyond its transcriptional regulation, we investigated the genomic context of TUBB2A. Analysis of somatic alterations revealed that samples with altered TUBB2A status were characterized by substantial genomic instability, evidenced by a high fraction of genome altered (FGA) and distinct patterns of copy number gains and losses (Figure 6D). Mutation profiling further identified missense mutations as the predominant variant type, with TUBB2A itself emerging as the most frequently mutated gene within the cohort (Figure 6E). Consistently, genome‐wide GISTIC analysis highlighted recurrent chromosomal amplifications and deletions across multiple loci (Figure 6F), indicating that the regulatory role of TUBB2A operates within a complex landscape of structural genomic variations.

## 4. Discussion

In the present study, by analyzing the gene expression profiles of AD patients comprehensively, we found that some inflammatory‐related genes (TUBB2A, RTN4, and YWHA) were robustly predictive in several external datasets. Analysis for the infiltration status of immune cells revealed significant alterations of Tregs, NK cells, and immune‐related genes such as PDCD1. With NMF, the tumors with AD were clustered into a group of high‐immune signature (Cluster C1), whereas low‐immune signature was Cluster C2. Pseudotemporal analysis mapped a developmental trajectory that began in healthy control and progressed through the C1 subtype, progressing into C2 stage indicating shift of AD pathology from inflammation to ER stress. All these results indicate that:

In line with previous reports, our results also support a key role for sustained neuroinflammation during AD progression, since microglial and astrocytic hyperactivity is associated with continuous production of proinflammatory factors including IL‐1*β*, TNF‐*α*, and IL‐6 [17–20]. Such cytokines alter the communication between neurons and induce neurodegenerative processes that exacerbate cognitive deficits. The fact that we found a downregulation for both TUBB2A as well as RTN4 in AD patients indicates that inflammation can cause changes to the cytoskeleton and synapses, suggesting that there may be a direct relationship between inflammation, cytokines, and brain structure integrity.

Our analysis of immune cell proportions reveals a complex, biphasic immune landscape in AD that challenges the traditional view of AD as a uniformly proinflammatory condition. Although theoretical models suggest that a loss of Tregs or NK cells predisposes individuals to autoimmunity or impaired pathogen clearance, our data uncover a more nuanced reality driven by disease progression: a paradoxical upregulation of immunosuppressive signatures (e.g., PDCD1) coexists with inflammatory markers, suggesting that chronic, overactive inflammation triggers a compensatory yet ultimately dysfunctional immunosuppressive response. This apparent contradiction underpins the significant immune heterogeneity we resolved through NMF, which delineated two distinct states: an overactive, proinflammatory state (C1) enriched in M1 macrophages and activated NK cells, and a hyporesponsive, exhausted state (C2) characterized by surging Tregs, quiescent NK cells, and elevated checkpoint markers. Rather than viewing the increase in regulatory cells in C2 merely as deleterious suppression, we interpret this as a hallmark of immune exhaustion—a state where the system, overwhelmed by persistent antigenic stress (e.g., A*β* and tau), shifts toward a tolerant but ineffective phenotype. This transition from the hyperinflammatory C1 to the hyporesponsive C2 supports an evolving model of AD immunopathogenicity where early immune activation eventually gives way to late‐stage immune paralysis, explaining the contradictory signatures observed in bulk analyses and highlighting the critical need for stage‐specific immunotherapies that target inflammation in C1 while potentially reversing immune exhaustion or restoring proteostasis in C2.

As a function of pseudotime, it allowed us to better understand how the different processes involved in inflammation during AD development evolve with time. In C1 cells, researchers found downregulation of genes involved in synapse function along with rising levels of inflammation indicators [21, 22]. Conversely, high AD patients (C2 class) had increased levels of ER stress genes including DDIT4 and AEBP1 [23–25], suggesting that the pathophysiology may involve an initial neuroinflammatory insult to neurons, which is then further exacerbated by the ER stress as well as other pathways of cellular stress. This time course suggests that the inflammatory response is not only an initial trigger, but also sustains subsequent pathogenic processes throughout progression of AD.

The detection of genes related to inflammation and immunity might be able to provide key biomarkers in early diagnosis and classification of AD. For example, it was shown that TUBB2A is an extremely good classifier [26], whereas PDCD1 and LGALS3 represent candidate targets for immunomodulatory therapies. Inflammation‐directed treatment—targeting the inflammatory cytokines such as IL‐1*β* and TNF‐*α* [17, 27], modulating immune checkpoint activity [28, 29], and directing therapies to microglial cells—seems effective at altering the disease course. Notably, such a molecular classification may lead toward the development of individualized therapies in which patients with distinct immunological profiles are best treated by specific therapeutic strategies.

Some limitations need to be pointed out in this work. First of all, we only conducted a transcriptomic analysis, requiring further confirmation in protein expression experiments as well as cell‐based function tests. Second, although ML improved the performance for predicting biomarkers, clinical evaluation on different patient cohorts is still required. Furthermore, considering the complexity and multifactorial nature of inflammation involved in AD, it seems likely that a combination of different omics would be needed, including proteomics and single cell analyses will be essential to understanding the full contribution of neuroinflammation to disease pathogenesis.

## Author Contributions

Wan Ma, Fenfang Zhou, Huaying Cai, and Ling Fa designed this work. Wan Ma, Fenfang Zhou, Huaying Cai, and Ling Fa drafted the manuscript. Wan Ma searched for relevant references and collected data from these studies. Wan Ma conducted experiments. Ling Fa interpreted results and made figures, who also contributed to editing of the final version of the manuscript. Ling Fa edited this paper.

## Funding

This study was supported by Natural Science Foundation of Zhejiang Province (10.13039/501100004731) (LY22H090017).

## Disclosure

All authors read and agreed to the final version.

## Ethics Statement

The authors have nothing to report.

## Conflicts of Interest

The authors declare no conflicts of interest.

## Supporting Information

Additional supporting information can be found online in the Supporting Information section.

## Supporting information


**Supporting Information 1** Figure S1: Functional enrichment analysis of DEGs. (A) GO analysis revealed enrichment in synaptic‐related processes, including “vesicle‐mediated transport in synapse” and “synaptic vesicle cycle.”(B) KEGG pathway analysis demonstrated significant enrichment in neuronal signaling and immune‐related pathways, suggesting a link between synaptic dysfunction and immune dysregulation in AD.

**Supporting Information 2** Figure S2: Pseudotime trajectory analysis of AD progression. (A) Pseudotime trajectory showing progression from controls → subtype C1 (early stage) → subtype C2 (late stage). (B) Heat map of dynamic gene expression across pseudotime, with synaptic function genes (RTN4 and TUBB2A) downregulated in early stages but upregulated later, whereas ER stress–related genes (DDIT4 and AEBP1) increased at late stages. These findings suggest a pathological transition from inflammation‐driven to ER stress–dominated mechanisms during AD progression.

## Data Availability

The data sets used and/or analyzed during the current study are available from the GEO repository, https://www.ncbi.nlm.nih.gov/geo/.
