# Supplementary figures and images for: Decoding Pathogenic Mutational Landscapes in Alzheimer′s Disease Through Integrated Transcriptomics
Source: Hum Mutat. 2026 May 12;2026:6627566. doi: 10.1155/humu/6627566 (PMC13162230; doi:10.1155/humu/6627566)

A Enriched pathways of top 50 marker genes

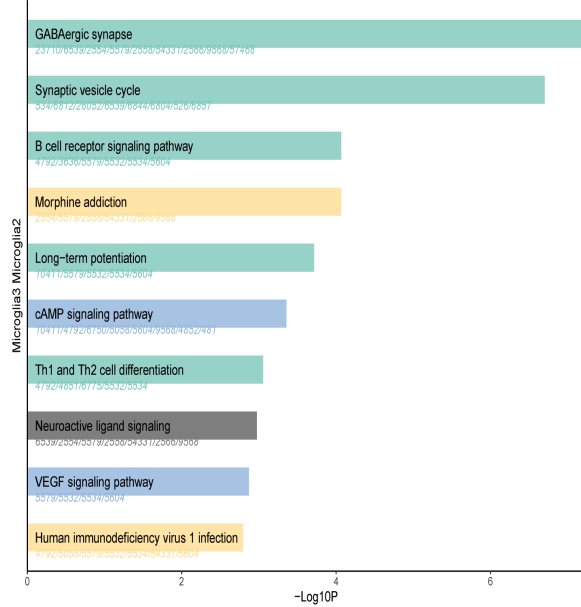

B

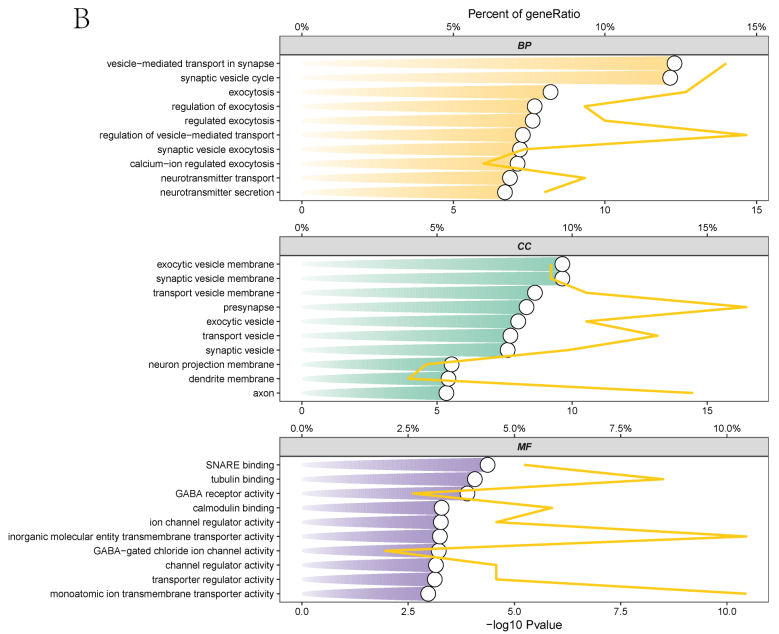

Supplement: Supplementary file 1 — Supporting Information 1 Figure S1: Functional enrichment analysis of DEGs. (A) GO analysis revealed enrichment in synaptic‐related processes, including “vesicle‐mediated transport in synapse” and “synaptic vesicle cycle.”(B) KEGG pathway analysis demonstrated significant enrichment in neuronal signaling and immune‐related pathways, suggesting a link between synaptic dysfunction and immune dysregulation in AD. [file HUMU-2026-6627566-s001.pdf]
