# Supplementary material for: Decoding Pathogenic Mutational Landscapes in Alzheimer′s Disease Through Integrated Transcriptomics
Source: Hum Mutat. 2026 May 12;2026:6627566. doi: 10.1155/humu/6627566 (PMC13162230; doi:10.1155/humu/6627566)
Supplement: Supplementary file 2 — Supporting Information 2 Figure S2: Pseudotime trajectory analysis of AD progression. (A) Pseudotime trajectory showing progression from controls → subtype C1 (early stage) → subtype C2 (late stage). (B) Heat map of dynamic gene expression across pseudotime, with synaptic function genes (RTN4 and TUBB2A) downregulated in early stages but upregulated later, whereas ER stress–related genes (DDIT4 and AEBP1) increased at late stages. These findings suggest a pathological transition from inflammation‐driven to ER stress–dominated mechanisms during AD progression. [file HUMU-2026-6627566-s002.pdf]

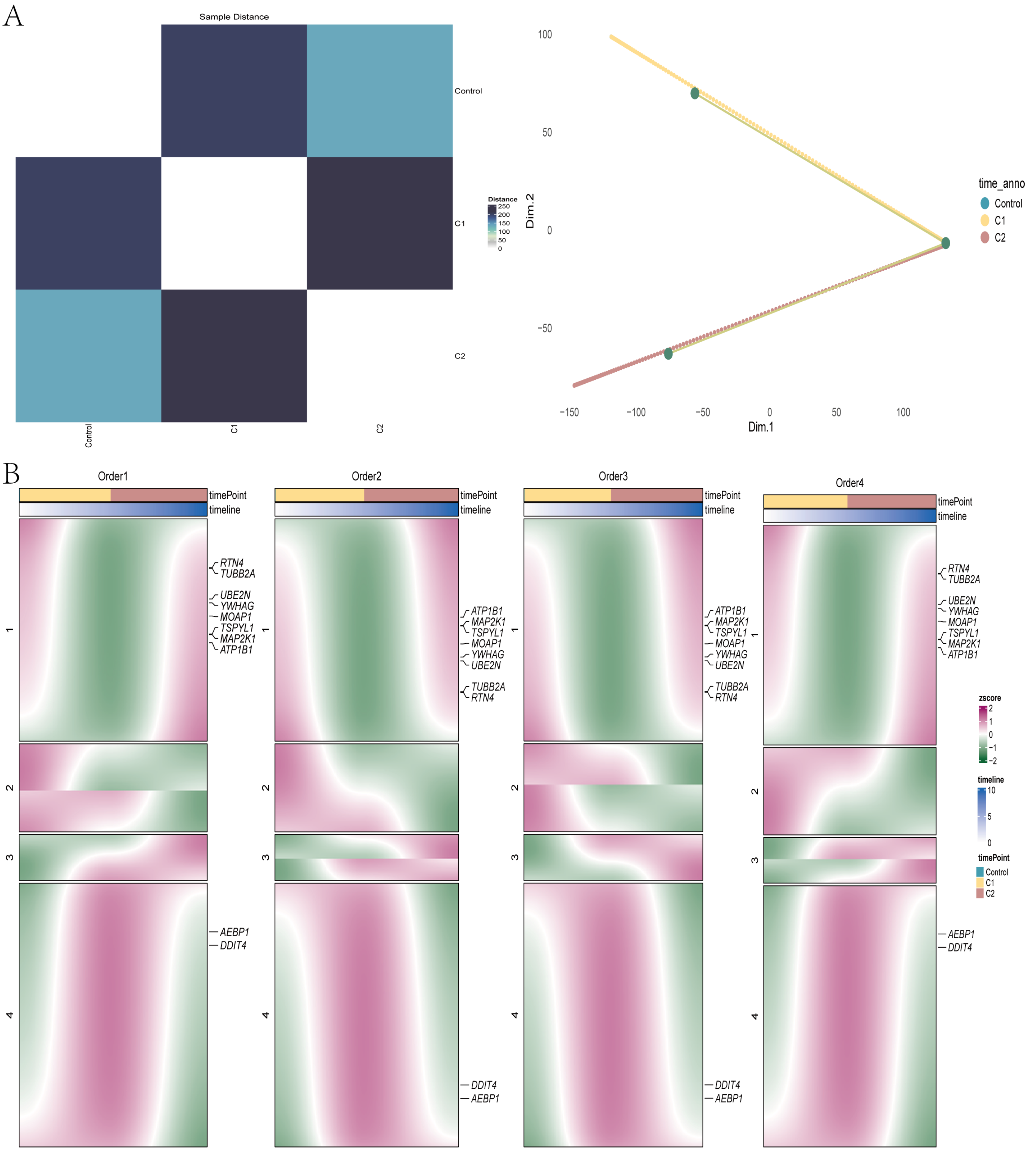

A
Sample Distance
Control
C1
C2
Distance
250
200
150
100
50
0
100
50
0
−50
−150
−100
−50
0
50
100
Dim.1
Dim.2
time_anno
Control
C1
C2
B
Order1
Order2
Order3
Order4
timePoint
timeline
RTN4
TUBB2A
UBE2N
YWHAG
MOAP1
TSPYL1
MAP2K1
ATP1B1
AEBP1
DDIT4
zscore
2
1
0
−1
−2
timeline
10
5
0
timePoint
Control
C1
C2
